# Acryl-3,5-bis(2,4-difluorobenzylidene)-4-piperidone targeting cellular JUN proto-oncogene, AP-1 transcription factor subunit inhibits head and neck squamous cell carcinoma progression

**DOI:** 10.37349/etat.2023.00184

**Published:** 2023-10-31

**Authors:** Levi Arnold, Juan Pineda Gomez, Michael Barry, Marrion Yap, Laura Jackson, Thuc Ly, David Standing, Subhash B. Padhye, Bernhard Biersack, Shrikant Anant, Sufi Mary Thomas

**Affiliations:** Fondazione IRCCS Istituto Nazionale dei Tumori, Italy; Nicola Normanno, Istituto Nazionale Tumori-IRCCS-Fondazione G. Pascale, Italy; ^1^Department of Cancer Biology, University of Kansas Medical Center, Kansas City, Kansas 66160, USA; ^2^Department of Otolaryngology, University of Kansas Medical Center, Kansas City, Kansas 66160, USA; ^3^Interdisciplinary Science and Technology Research Academy, University of Pune, Pune 411007, Maharashtra, India; ^4^Department of Biology, Chemistry, Earth Sciences, University of Bayreuth, 95440 Bayreuth, Germany

**Keywords:** Head and neck squamous cell carcinoma, JUN proto-oncogene, transcription factor, small molecule inhibitor

## Abstract

**Aim:**

Head and neck squamous cell carcinoma (HNSCC) is the seventh most common cancer worldwide with a survival rate below fifty percent. Addressing meager therapeutic options, a series of small molecule inhibitors were screened for antitumor efficacy. The most potent analog, acryl-3,5-bis(2,4-difluorobenzylidene)-4-piperidone (DiFiD; A-DiFiD), demonstrated strong cellular JUN proto-oncogene, activator protein 1 (AP-1) transcription factor subunit (JUN, c-Jun) antagonism. c-Jun, an oncogenic transcription factor, promotes cancer progression, invasion, and adhesion; high (*JUN*) mRNA expression correlates with poorer HNSCC survival.

**Methods:**

Four new small molecules were generated for cytotoxicity screening in HNSCC cell lines. A-DiFiD-treated HNSCC cells were assessed for cytotoxicity, colony formation, invasion, migration, and adhesion. Dot blot array was used to identify targets. Phospho-c-Jun (p-c-Jun) expression was analyzed using immunoblotting. The Cancer Genome Atlas (TCGA) head and neck cancer datasets were utilized to determine overall patient survival. The Clinical Proteomic Tumor Analysis Consortium (CPTAC) datasets interfaced with University of Alabama at Birmingham Cancer Data Analysis Portal (UALCAN) were analyzed to determine protein levels of c-Jun in HNSCC patients and correlate levels with patient.

**Results:**

Of the small molecules tested, A-DiFiD was the most potent in HNSCC lines, while demonstrating low half-maximal drug inhibitory concentration (IC_50_) in non-malignant Het-1A cells. Additionally, A-DiFiD abrogated cell invasion, migration, and colony formation. Phospho-kinase *in vitro* array demonstrated A-DiFiD reduced p-c-Jun. Likewise, a time dependent reduction in p-c-Jun was observed starting at 3 min post A-DiFiD treatment. TCGA Firehose Legacy *vs. *recurrent and metastatic head and neck cancer reveal a nearly 3% DNA amplification in recurrent/metastatic tumor compared to below 1% in primary tumors that had no lymph node metastasis. CPTAC analysis show higher tumor c-Jun levels compared to normal. Patients with high JUN expression had significantly reduced 3-year survival.

**Conclusions:**

A-DiFiD targets c-Jun, a clinical HNSCC driver, with potent anti-tumor effects.

## Introduction

Head and neck squamous cell carcinoma (HNSCC) is the seventh most common cancer worldwide, contributing to 325,000 deaths and 660,000 new cases every year [1]. Despite recent therapeutic advances, the five-year survival remains below 50% [2]. There is an urgent need for development of effective small molecule inhibitors [3]. The analogues of 3,5-bis(2,4-difluorobenzylidene)-4-piperidone (DiFiD) for antitumor effects in HNSCC. Acryl-DiFiD (A-DiFiD), the most cytotoxic analog, was discovered to target cellular JUN proto-oncogene, activator protein 1 (AP-1) transcription factor subunit (JUN, c-Jun).

c-Jun is the first oncogenic transcription factor discovered that is encoded by the *JUN* gene and the cellular derivative of viral oncoprotein sarcoma virus 17 oncogene homolog avian (v-Jun) [4, 5]. c-Jun in conjunction with cellular-Fos (c-Fos) are AP-1 family transcription factors acting on immediate early response genes [6]. c-Jun activation is generally achieved through the mitogen-activated protein kinase (MAPK) pathway, stimulated by many factors including cytokines, reactive oxygen species, ultraviolet radiation, and other protein factors like the epidermal growth factor (EGF) [7–10].

Activation of c-Jun increases the transcription of target genes that play a role in apoptosis, differentiation, cellular division, proliferation, and cell survival [11, 12]. Clinically c-Jun over expression has been noted in non-small cell lung cancers (NSCLCs) [13], vulvar squamous cell carcinoma [14], colorectal cancer [15], Hodgkin lymphoma [16], and recently in HNSCC [17]. Further, c-Jun has been reported to promote metastases and invasion in several tumors including breast, skin, liver, fibrosarcoma and HNSCC [18–22]. In HNSCC, c-Jun expression drives poor differentiation and aggressive tumorigenesis, while correlating with an increased expression of the pro-invasive factor matrix metalloprotease 9 (MMP9) [23].

Upregulation of c-Jun contributes to docetaxel, cisplatin, and 5-fluorouracil drug resistance [24]. Further, short hairpin RNA silencing of c-Jun has been shown to enhance response of HNSCC cells to phosphatidylinositol-4,5-bisphosphate 3-kinase catalytic subunit alpha (PI3Kα) inhibitor alpelisib [25]. The small molecule SP600125 has been shown to competitively bind c-Jun in peripheral blood mononuclear cells (PBCs) [26]. Additionally, the inhibitor CC-90001 is in clinical investigation for the therapeutic response of idiopathic pulmonary fibrosis [27]. There are no reports testing small molecule inhibitors of c-Jun in HNSCC. To address this gap, the efficacy of A-DiFiD and other c-Jun inhibitors on HNSCC was determined. This report demonstrates that c-Jun is a therapeutic target for HNSCC and that A-DiFiD inhibits c-Jun phosphorylation, further advancing targeted therapies for HNSCC.

## Materials and methods

### Cells and reagents

Well characterized HNSCC cells were used throughout this study [28]. HNSCC lines UMSCC1 (a gift from Dr. Tom Carey, University of Michigan, Ann Arbor, MI) , HN5 (a gift from Dr. Jeff Myers, The University of Texas MD Anderson Cancer Center, Houston, TX), OSC19 (a gift from Theresa Whiteside, University of Pittsburgh, Pittsburgh, PA), Cal33 (cat# ACC 447 DSMZ; Braunschweig, Germany), and FaDu (cat# HTB-43, ATCC; Gathersburg, MD, USA) were maintained in high glucose Dulbecco’s Modified Eagle Medium (DMEM; Corning, NY, USA) containing 10% heat-inactivated fetal-bovine serum (Neuromics, MN, USA). Non-cancerous, immortalized esophageal Het-1A (cat# CRL-2692, ATCC; Gathersburg, MD, USA) line was maintained in bronchial epithelial cell growth basal medium supplemented with bronchial epithelial cell growth medium (BEGM) kit (cat# CC-4175, Lonza, Basel, Switzerland). Cisplatin (cat# 4015472, Fresenius Kabi, Lake Zurich, IL, USA), CC-90001 (cat# HY-138304, Med Chem Express, Monmouth Junction, NJ, USA), SP600125 (cat# 420119, EMD Millipore, Burlington, MA, USA).

### Chemistry

In general, melting points (m.p., uncorrected) were determined using Electrothermal 9100 (cat# 03010-51, model: Electrothermal IA9100, TEquipment, Long Branch, NJ, USA). Infrared (IR) spectra was determined using Perkin-Elmer Spectrum One FT-IR spectrophotometer (cat# FTIR-01-0240, model: Spotlight 300 FT-IR MCT, Shelton, CT, USA) with attenuated total reflectance (ATR) sampling unit. For the nuclear magnetic resonance (NMR) spectra we used Bruker Avance 300/500 spectrometer (cat# DIS-46950, model: Avance 500, Billerica, MA, USA). Chemical shifts (δ) in parts per million (ppm) downfield from tetramethylsilane (internal standard) and mass spectra we calculated using Thermo Finnigan MAT 8500 (EI; cat# 8500, model: 2000FRM, Waltham, MA, USA).

#### A-DiFiD

DiFiD (92 mg, 0.27 mmol) was suspended in acetone and treated with acryloyl chloride (35 µL, 0.43 mmol; cat# 549797, EMD Millipore, Burlington, MA, USA). K_2_CO_3_ (cat# 584-08-7, EMD Millipore, Burlington, MA, USA; 197 mg, 1.43 mmol, dissolved in 2 mL H_2_O) was added and the reaction mixture was stirred at room temperature for 24 h. Water (20 mL) was added and the formed precipitate was collected, washed with water and dried in vacuum. Yield: 70 mg (0.17 mmol, 64%); yellow solid of m.p. 192°–193°C; *υ*_max_(ATR)/cm^–1^ 3,070, 1,675, 1,652, 1,613, 1,579, 1,497, 1,468, 1,447, 1,426, 1,344, 1,308, 1,270, 1,239, 1,219, 1,182, 1,166, 1,147, 1,130, 1,096, 986, 962, 928, 907, 888, 861, 829, 813, 788, 750, 742, 730, 699, 679, 614; ^1^H NMR (300 MHz, CDCl_3_) *δ* 4.6–4.8 [4 H, minute (m)], 5.5–5.6 (1 H, m), 6.1–6.3 (2 H, m), 6.8–7.0 (4 H, m), 7.2–7.4 (2 H, m), 7.81 [2 H, second (s)]; ^13^C NMR (75.5 MHz, CDCl_3_) *δ* 43.8, 46.7, 104.4–105.1 (m), 111.8–112.1 (m), 118.6, 126.4, 129.1, 129.9, 131.7, 131.8, 133.1, 159.5, 162.0–162.2 (m), 162.8, 165.4–165.5 (m), 185.6; mass/charge (m/z, %), 401 (100) [M^+^], 346 (37), 151 (56), 55 (57).

#### Cl-DiFiD

1-(4-Chlorobenzenesulfonyl)-4-piperidinone (cat# 130036, EMD Millipore, Burlington, MA, USA; 216 mg, 0.79 mmol) and 2,4-difluorobenzaldehyde (cat# 265179, EMD Millipore, Burlington, MA, USA; 213 mg, 1.5 mmol) were dissolved in ethanol (EtOH; 5 mL; cat# E7023-1L, EMD Millipore, Burlington, MA, USA) and conc. Hydrochloric acid (HCl; 1 mL; cat# 1373122500, EMD Millipore, Burlington, MA, USA) was added. The reaction mixture was refluxed for 24 h. After cooling down to room temperature, the formed yellow precipitate was collected, washed with EtOH and dried in vacuum. Yield: 200 mg (0.38 mmol, 49%); yellow solid of m.p. 270°–272°C; *υ*_max_(ATR)/cm^–1^ 3,100, 1,612, 1,587, 1,499, 1,476, 1,461, 1,426, 1,381, 1,356, 1,342, 1,271, 1,232, 1,165, 1,138, 1,091, 1,074, 1,053, 1,028, 1,014, 987, 967, 957, 918, 880, 857, 843, 831, 821, 809, 778, 756, 726, 709, 677, 647, 637, 614, 608; ^1^H NMR [300 MHz, dimethylsulfoxide (DMSO)-d_6_] *δ* 3.4–3.9 (3 H, m), 4.3–4.5 (1 H, m), 6.3–6.5 (1 H, m), 6.7–6.9 (1 H, m), 7.1–7.7 (9 H, m), 7.8–7.9 (1 H, m); ^13^C NMR (75.5 MHz, DMSO-d_6_) *δ* 43.2, 44.6, 45.9, 46.4, 47.7, 48.1, 95.6, 102.9–105.1 (m), 106.1, 112.1–112.4 (m), 115.7, 118.1, 122.1, 124.1, 126.8, 128.8–129.7 (m), 131.9, 132.3–132.5 (m), 135.3, 135.9, 136.4, 138.3–138.5 (m), 140.6, 150.5–150.7 (m), 158.8, 160.7, 161.4, 161.6, 164.9, 183.4; m/z (%), 521 (67) [M^+^], 346 (100), 317 (37), 298 (17), 194 (22), 180 (56), 152 (88), 127 (21).

#### MeO-DiFiD

1-(4-Methoxybenzenesulfonyl)-4-piperidinone (cat# 8.20828, EMD Millipore, Burlington, MA, USA; 213 mg, 0.79 mmol) and 2,4-difluorobenzaldehyde (213 mg, 1.5 mmol) were dissolved in EtOH (10 mL) and conc. HCl (1 mL) was added. The reaction mixture was refluxed for 24 h. After cooling down to room temperature, the formed precipitate was collected, washed with EtOH and dried in vacuum. Yield: 125 mg (0.24 mmol, 30%); yellow solid of m.p. 251°–252°C; *υ*_max_(ATR)/cm^–1^ 3,080, 2,842, 1,595, 1,498, 1,462, 1,428, 1,340, 1,309, 1,261, 1,181, 1,153, 1,091, 1,072, 1,025, 987, 967, 917, 882, 836, 804, 775, 753, 730, 697, 670, 627, 610; ^1^H NMR (300 MHz, DMSO-d_6_) *δ* 3.4–3.9 (6 H, m), 4.3–4.5 (1 H, m), 6.3–6.5 (1 H, m), 6.8–6.9 (1 H, m), 7.0–7.5 (8 H, m), 7.6–7.7 (1 H, m), 7.7–7.8 (1 H, m); ^13^C NMR (75.5 MHz, DMSO-d_6_) *δ* 42.9, 44.7, 45.8, 47.8, 48.2, 55.7, 95.7, 102.9–104.3 (m), 106.2, 109.2–109.5 (m), 111.6, 114.4–114.7 (m), 115.8, 119.1, 122.0, 124.3, 127.3, 127.7, 128.2–128.5 (m), 129.4–129.6 (m), 131.5, 140.6, 150.5–150.7 (m), 158.8, 160.6, 162.9; m/z (%), 517 (7) [M^+^], 486 (6), 346 (100), 201 (68), 171 (26), 108 (22).

#### DF-DiFiD

1-(3,4-Difluorobenzenesulfonyl)-4-piperidinone (cat# 555913, EMD Millipore, Burlington, MA, USA; 217 mg, 0.79 mmol) and 2,4-difluorobenzaldehyde (213 mg, 1.5 mmol) were dissolved in EtOH (10 mL) and conc. HCl (1 mL) was added. The reaction mixture was refluxed for 24 h. After cooling down to room temperature and 24 h in the refrigerator, the formed yellow crystals were collected, washed with EtOH and dried in vacuum. Yield: 152 mg (0.29 mmol, 37%); yellow solid of m.p. 273°–274°C; *υ*_max_(ATR)/cm^–1^ 3,066, 1,603, 1,542, 1,498, 1,463, 1,417, 1,382, 1,343, 1,316, 1,278, 1,247, 1,212, 1,163, 1,139, 1,092, 1,070, 1,028, 1,009, 987, 968, 955, 914, 879, 857, 844, 823, 774, 753, 741, 731, 716, 697, 674, 654, 632, 611; ^1^H NMR (300 MHz, DMSO-d_6_) *δ* 3.5–4.0 (3 H, m), 4.3–4.6 (1 H, m), 6.3–6.5 (1 H, m), 6.8–6.9 (1 H, m), 7.1–7.4 (5 H, m), 7.5–7.8 (3 H, m), 7.8–7.9 (1 H, m).

### Cytotoxicity assay

Cells (4 × 10^3^ cells/well, 96-well plates; cat# 229196, Cell Treat, Pepperel, MA, USA) were treated in quadruplicate with A-DiFiD or indicated compounds at various concentrations, and cell proliferation assessed using CyQUANT assay kit (Life Technologies, Waltham, MA, USA) per manufacturer’s instructions at 72 h. Half-maximal drug inhibitory concentration (IC_50_) was calculated using non-linear curve fit with GraphPad Prism software version 9.0 (GraphPad Software Inc., San Diego, CA, USA).

### Colony formation assay

A total of 300 HNSCC cells pretreated with predetermined doses of A-DiFiD (IC_50_) for 30 min then seeded in a 6-well plate (cat# 229105, Pepperel, MA, USA). Drug-free cell culture medium was replaced every 2–3 day. When colony visibility appeared in one of the 6-well plates (10–14 days), the culture was terminated by fixing with 10% formalin (cat# HT501128-4L, Sigma Aldrich, St. Louis, USA) for 30 min. Plates were stained with crystal violet (cat# C6158 Sigma Aldrich, St. Louis, USA) for 10 min at room temperature then washed with water 3 times. The number of cell colonies was counted under the microscope (< 75 clones validated). Formed colonies are determined by visualizing at least 50 cell per cluster.

### Migration and invasion assay

Cells (2 × 10^4^ cells per insert) were transferred to trans-well inserts with 8 μm pores (Becton Dickinson, Franklin Lakes, NJ, USA). For invasion assay, a layer of diluted Matrigel (2 mg/mL at 100 µL volume) in DMEM (BD Biosciences, San Jose, CA, USA) was placed in the insert. Cells in serum-free media were seeded onto Matrigel layer for invasion or directly onto insert for migration assay. The inserts were placed in duplicate holding-wells containing A-DiFiD at half IC_50_ in complete media for 24 h. Treated and untreated cells were plated in parallel to assess viability using CyQuant assay kit. The number of cells that moved to other side of membrane were counted following fixation and staining using Hema3 kit (Fisher Scientific, Hampton, NH, USA). The numbers of migrating or invading cells were normalized to cell viability.

### Cell adhesion and spread assay

A total of 6 × 10^5^ HNSCC cells were pretreated with A-DiFiD (IC_50_) for 30 min then plated on 60 mm dishes coated with collagen (cat# A1048301, Thermo, Waltham, MA, USA), fibronectin (cat# 33016015, Thermo), vitronectin (cat# A14700, Thermo, Waltham, MA, USA), or laminin (cat# L4544, Sigma Aldrich, St. Louis, USA). Cells were allowed to adhere for 15, 30, 60, or 120 min before the plates were washed with 10 mL phosphate-buffered saline (PBS), then stained with crystal violet (cat# C6158 Sigma Aldrich, St. Louis, USA). Images were taken under the microscope and analyzed for number of adherent cells per plate as well as cell area (cell spread). Spread and adhesion were quantified using ImageJ (version 13.0.6 National Institutes of Health, Bethesda, MD, USA).

### Human phospho-kinase array

Proteome Profiler Human Phospho-Kinase Array Kit (ARY003C, R&D Systems, Minneapolis, MN, USA) was used to identify signaling targets of A-DiFiD per the manufacturer’s instructions. Membranes were image by autoradiography, and ImageJ was used to quantify the signal intensity.

### Immunoblotting

Cells (3 × 10^5^ cells/60 mm dish) were treated with A-DiFiD (IC_50_) and lysed in radioimmunoprecipitation assay (RIPA) buffer containing protease/phosphatase inhibitors (complete, Mini, Roche, Indianapolis, IN, USA). For inhibition of phospho-c-Jun (p-c-Jun) stimulation, cells were serum starved for 48 h, and then treated with A-DiFiD (IC_50_) for 0, 3, 5, 10, 20, 40, or 80 min. Cell lysates were harvested on ice and proteins separated by sodium dodecyl-sulfate polyacrylamide gel electrophoresis (SDS-PAGE), transferred onto nitrocellulose membranes, and probed with p-c-Jun (R&D Systems, Minneapolis, MN, USA) or pan-actin (Cell Signaling, Danvers, MA, USA). Protein bands were detected using anti-mouse or anti-rabbit immunoglobulin G (IgG) conjugated to Dylight-680 or Dylight-800 (Fisher, Waltham, MA, USA) and quantified using Li-Cor Odyssey protein imaging system (Li-Cor Biotechnology, Lincoln, NE, USA).

### Analysis of The Cancer Genome Atlas

Using the cBioPortal [29, 30] interface, DNA amplification data was generated by probing two The Cancer Genome Atlas (TCGA) datasets: HNSCC Firehose Legacy (512 samples total, 497 samples primary tumor only), and recurrent and metastatic (151 samples total, 56 HNSCC samples selected) datasets which include 151 samples from patients with advanced, treatment-resistant head and neck tumors that are characterized as recurrent and/or metastatic [31]. *JUN* DNA copy number amplification, deletion, or mutation were assessed, and percent amplification is expressed as percent of whole. *JUN* mRNA (RNA Seq V2) was utilized from TCGA Firehose Legacy (512 samples) to generate comparative analysis between *JUN* levels in either normal or tumor samples. To generate survivorship comparison, TCGA Firehose Legacy databank was assessed by log-rank (Mantel-Cox) text assessing differences between curves. Patients were stratified into two groups and correlation between reads per kilobase [reads per kilobase million (RPKM)] and survival were evaluated. RPKM values were stratified by into high/low cutoffs of 0.5.

### Clinical Proteomic Tumor Analysis Consortium analysis

Clinical Proteomic Tumor Analysis Consortium (CPTAC) database was accessed using the interface of University of Alabama at Birmingham Cancer Data Analysis Portal (UALCAN) [32, 33]. To evaluate tumor *vs.* normal, normal *vs.* tumor staging, normal *vs.* tumor patient weight, and normal *vs.* gender plots were generated using *Z*-values that represent standard deviations from the median across the tumor. Log2 spectral count ratio values from CPTAC were initially normalized within each sample profile, then normalized across samples.

### Statistical analysis

Data are reported as mean ±  standard error of the mean (SEM). Nonparametric Mann-Whitney test utilized to evaluate significance in all experiments unless otherwise stated. For TCGA survivorship comparison, log-rank (Mantel-Cox) test evaluated differences between curves. Expression cut off was generated by stratifying patients into two groups and association between survival and RPKM was assessed. RPKM values in the highest *vs.* lowest quartiles was used and *P*-values determined. All statistical calculations were performed on GraphPad Prism software (version 9.0), with significance determined by *P* < 0.05.

## Results

### A-DiFiD demonstrates potent HNSCC cytotoxicity

**Figure 1:**
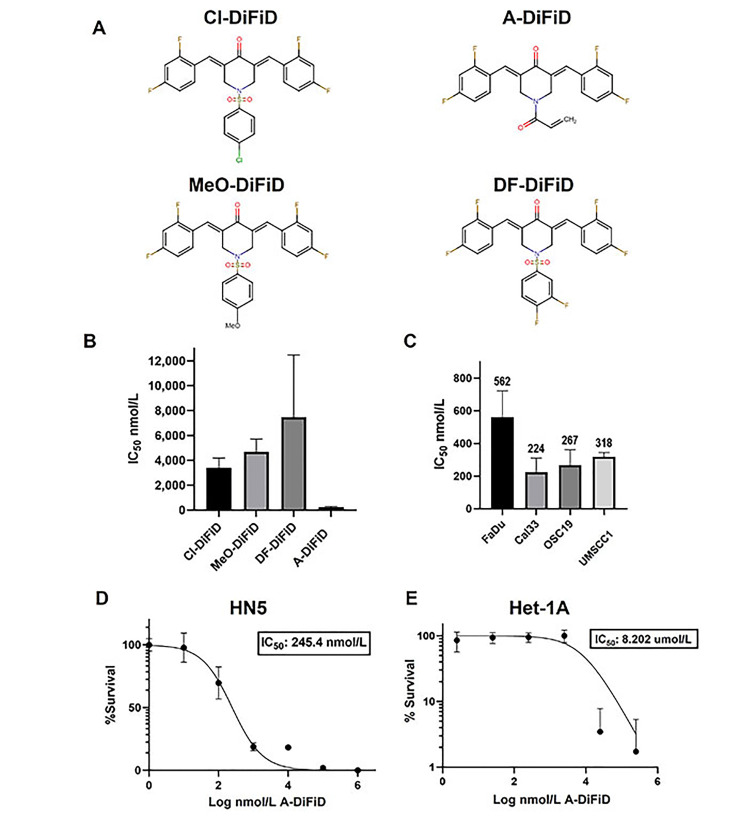
A-DiFiD demonstrates cytotoxic activity against HNSCC. (A) Chemical structure of four DiFiD analogs Cl-DiFiD, A-DiFiD, MeO-DiFiD, and DF-DiFiD; (B) HN5 (4 × 10^3^ cells/well in triplicate) were treated with various concentrations of Cl-DiFiD, A-DiFiD, MeO-DiFiD, and DF-DiFiD; (C) A-DiFiD IC_50_ determination against HNSCC cell lines FaDu, Cal33, OSC19, and UMSCC1; (D) A-DiFiD IC_50_ on HN5 HNSCC cell line expressed by percent survival; (E) Het-1A treated with various doses of A-DiFiD. IC_50_ was calculated using non-linear curve fit from three separate experiments on GraphPad Prism software

Small molecule inhibitor DiFiD, has antitumor effects in several cancers [3, 34]. To enhance the antitumor efficacy of DiFiD, we developed four analog small molecule compounds. We tested the cytotoxicity of all four compounds using serial dilutions (1:1, 1:10, 1:100, 1:1 × 10^3^, 1:1 × 10^4^, 1:1 × 10^5^, 1:1 × 10^6^) and DMSO control. A-DiFiD demonstrated the greatest cytotoxicity (HN5 IC_50_: 245.4 nmol/L) compared to Cl-DiFiD (HN5 IC_50_: 3.393 µmol/L), MeO-DiFiD (HN5 IC_50_: 4.690 µmol/L), and DF-DiFiD (HN5 IC_50_: 7.480 µmol/L) (Figure 1A and B). A-DiFiD demonstrated nanomolar toxicity in FaDu, Cal33, OSC19, and UMSCC1 HNSCC cells lines (Figure 1C and D).

A-DiFiD (HN5 IC_50_ nmol/L) has comparable toxicity to CC-90001 (HN5 IC_50_ 200.4 nmol/L) in HNSCC cells (Figure S1A). Additionally, A-DiFiD had a greater cytotoxicity than SP600125 (HN5 IC_50_ 523.7 nmol/L) (Figure S1B). These results demonstrate A-DiFiD has comparable if not more potent cytotoxic effects comparted to other c-Jun inhibitors available commercially. A major barrier in the development and clinical application of small molecules is the exposure of non-cancerous cells and tissue to cytotoxic effects. To address this concern, we treated Het-1A, an immortalized esophageal squamous cell line from a cancer free patient, with A-DiFiD and observed a many fold increase in IC_50_ (8.202 µmol/L) compared to HNSCC lines (Figure 1E). This indicates that A-DiFiD is well tolerated in normal cells.

### A-DiFiD abrogates HNSCC proliferation, migration, invasion, and adhesion *in vitro*

**Figure 2:**
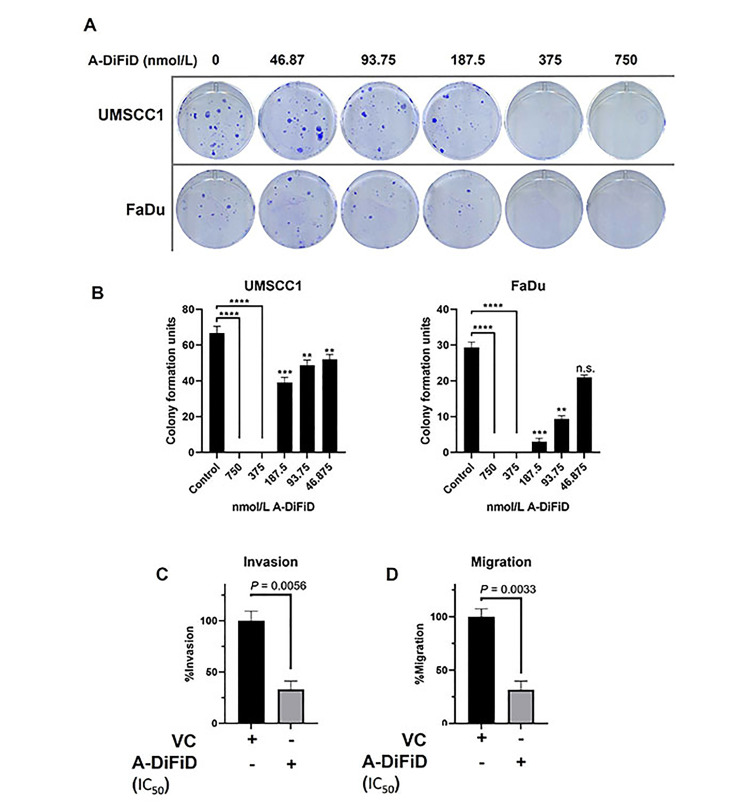
A-DiFiD (IC_50_, 4h) treatment abrogates HNSCC colony formation, migration, and invasion. (A) Representative image of colony formation of UMSCC1 and FaDu; (B) quantification of colony formation. Data represents cumulative results from two independent experiments with mean ± SEM; (C) invasion; and (D) migration are normalized for differences in proliferation rates over the duration of the experiment. Data represents cumulative results from three independent experiments with mean ± SEM. ^**^ *P* < 0.01, ^***^ *P* < 0.001, ^****^ *P* < 0.0001; n.s.: not significant; VC: vehicle control

For cancer cells to successfully form a tumor, they must first acquire the ability for a single cancer cell to adhere to a substrate, replicate, and form a colony. To test the ability of a single HNSCC cell to replicate when challenged with A-DiFiD, we performed colony formation assay. HNSCC cells were collected by trypsinization and washed several times with PBS and then held in a suspension of DMEM with various concentrations of A-DiFiD for 30 min. Cells were then washed again and only 300 cells were plated per well in a 6-well dish containing drug-free culture medium. HNSCC cell lines pretreated A-DiFiD demonstrated significantly reduced ability to proliferate (Figure 2A and B).

No treatment options for the metastatic spread of HNSCC are available. Further, patients with metastatic spread have an abysmal survival rate of less than 20% [35]. For cancer to metastasize, a local tumor must first break down the extracellular matrix (ECM) that confines it to a local space. To test this, we performed a trans-well invasion assay wherein HNSCC cells must move through a layer of Matrigel before they can be stained and quantified. HNSCC cells treated with IC_50_ A-DiFiD demonstrated a significant reduction in invasion (Figure 2C). Another important feature of successful tumor spread is the ability for cells to migrate. To test this, we utilized the same transwell assay as before, but no Matrigel was added. We observed that HNSCC cell migration was significantly reduced when treated with A-DiFiD (Figure 2D).

**Figure 3:**
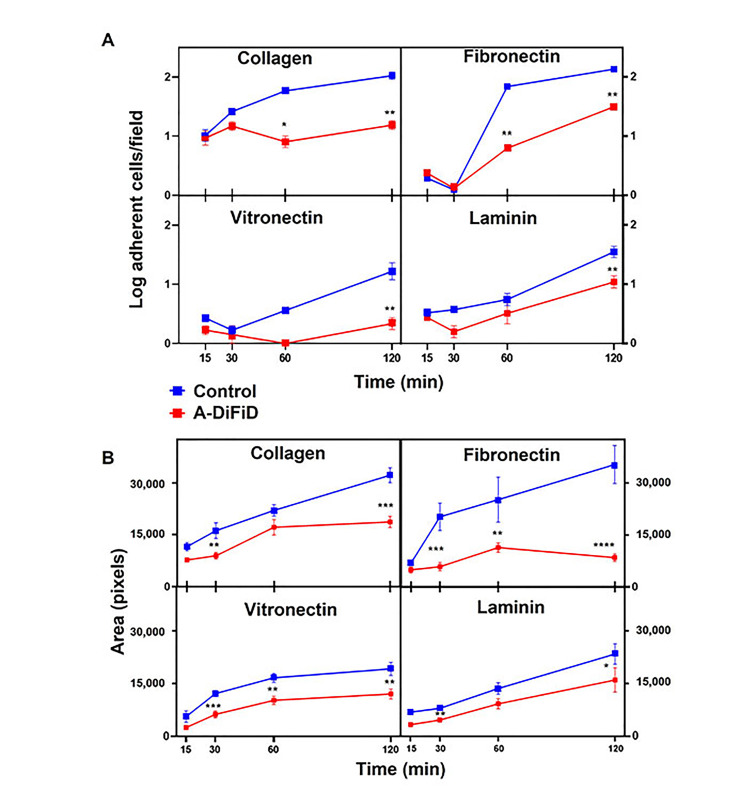
HNSCC adherence and spread is reduced by A-DiFiD (IC_50_, 30 min) pretreatment. Collagen, fibronectin, vitronectin, and laminin. (A) Adherent cells per field; and (B) cell pixel area were counted over various time points. Data represent cumulative results from three independent experiments with mean ± SEM. ^*^ *P* < 0.05, ^**^ *P* < 0.01, ^***^ *P* < 0.001, ^****^ *P* < 0.0001

Cell adhesion is important for squamous cell carcinoma to participate in cell-ECM interactions, and plays a crucial role in the progression, recurrence, invasion, and distant metastasis [36]. To test the ability of HNSCC challenged with A-DiFiD treatment to adhere to ECM, we employed a cell adhesion assay. Cells were plated on collagen, fibronectin, vitronectin, or laminin and allowed to adhere for various time points before being washed, stained, and quantified. We found that A-DiFiD reduced the HNSCC cellular adhesion on collagen, fibronectin, vitronectin, and laminin ECM in a time dependent manner up to 120 min (Figure 3A). Cell adhesion is typically followed by activation of intracellular signaling pathways that regulated cell spreading over the ECM. We also measured the surface area occupied by individual cells to determine if A-DiFiD impacts cell spreading. We found that A-DiFiD treatment reduced the surface area occupied by cells on all ECM substrates we tested (Figure 3B).

### A-DiFiD targets c-Jun

**Figure 4:**
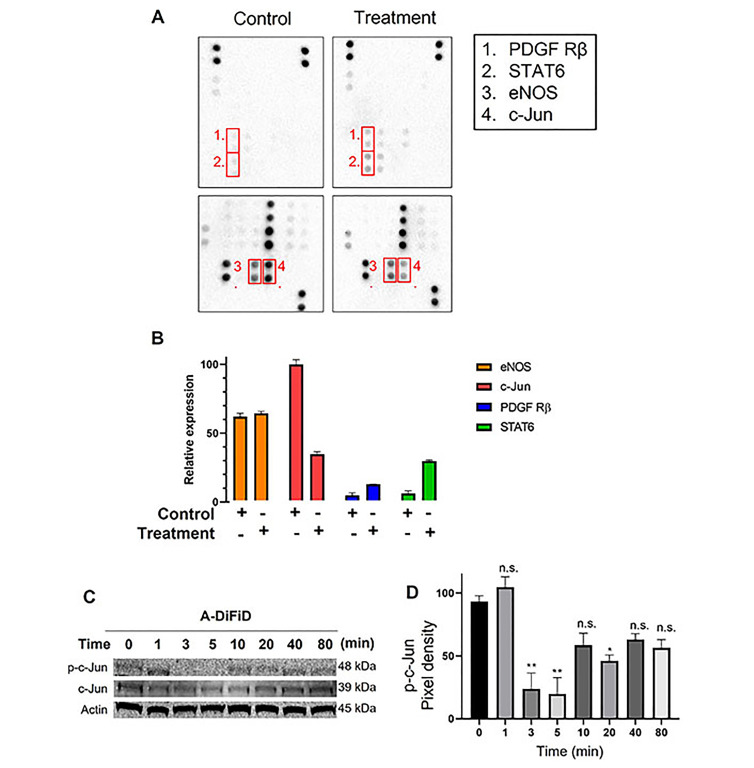
c-Jun is the molecular target of A-DiFiD. (A) HNSCC cells (FaDu; 2 × 10^5^ cells) were treated with vehicle control or IC_50_ concentrations of A-DiFiD for 24 h. Representative dot-blot images from phospho-kinase array; (B) densitometric analysis of array signal from A-DiFiD treated lysate normalized to those treated with vehicle control and represented as fold change in protein levels; (C) HNSCC cells (FaDu; 2 × 10^5^ cells) were treated with IC_50_ concentrations of A-DiFiD and protein lysate collected over various time points. Image is representative of three independent experimental repeats; (D) densitometric analysis of signals from immunoblot normalized to loading control. Error bars represent ± SEM. ^*^ *P* < 0.05, ^**^ *P* < 0.01

HNSCC cells exploit a multitude of molecular pathways that regulate growth and metastasis. To assess the mechanism of action of A-DiFiD, we used a phospho-protein array to identify the molecules perturbed by A-DiFiD treatment. Phosphorylation of c-Jun was the most significantly reduced in HNSCC treated with A-DiFiD treatment (Figure 4A and B). Further, we validated a reduction in p-c-Jun levels as early as at 3 min post-treatment (Figure 4C and D, Figure S2A and B). These data demonstrate that A-DiFiD targets c-Jun, an important molecular mediator in the progression of HNSCC.

### c-Jun drives HNSCC progression

**Figure 5:**
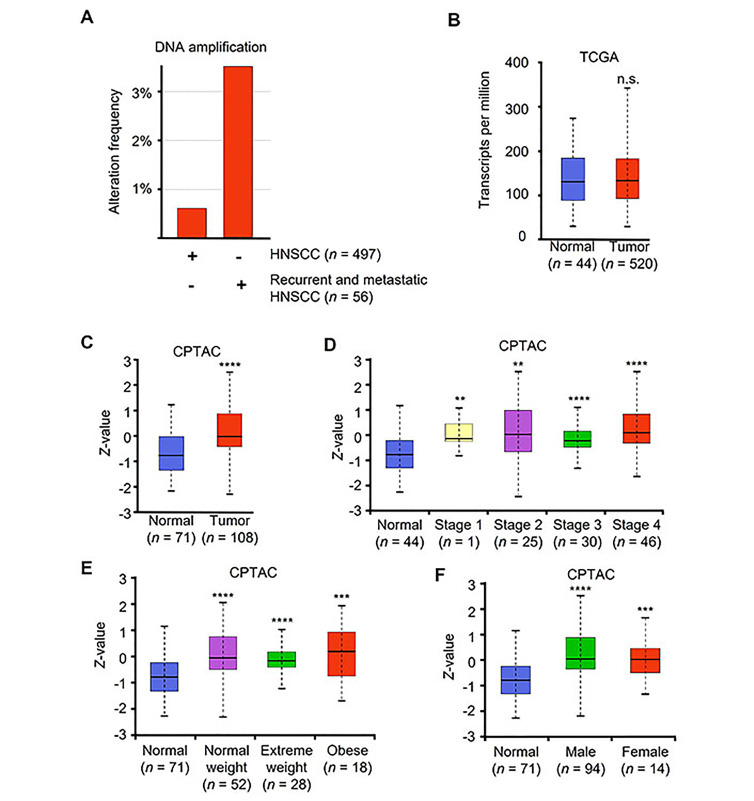
HNSCC patient tumor samples have increased levels of c-Jun. (A) Alteration frequency of two independent datasets, TCGA Firehose Legacy and MSK recurrent and metastatic head and neck cancer. DNA copy number alteration and amplification shown as percent of whole data set copy number alteration frequency; (B) *JUN* mRNA levels from TCGA (Firehose Legacy) as shown in transcripts per million; (C) c-Jun protein levels of normal *vs.* tumor from CPTAC head and neck cancer dataset shown using *Z*-value score; (D) c-Jun protein levels comparing normal to HNSCC stages 1–4; (E) normal *vs.* tumor at various patient weight, and (F) normal *vs.* tumor patient gender. ^**^ *P* < 0.01, ^***^ *P* < 0.001, ^****^ *P* < 0.0001

Patients with recurrent or metastatic HNSCC have significantly worse prognosis. Compared to primary HNSCC, metastatic and recurrent HNSCC patient samples had a significant increase in *JUN* DNA copy number amplification (Figure 5A). Interestingly, mRNA levels of *JUN* were not significantly different between normal and tumor samples (Figure 5B).

CPTAC provides proteomic data across a variety of cancer types. CPTAC dataset for HNSCC contains 108 tumor samples and 71 normal samples. Using UALCAN to access HNSCC CPTAC dataset, we found that protein levels were significantly elevated in patient tumor samples compared to normal samples (Figure 5C). Additionally, all tumor stages demonstrated significantly elevated protein compared to normal (Figure 5D). We also noted that all patients regardless of weight or gender had elevated c-Jun protein levels compared to normal tissue (Figure 5E and F).

The treatment outcomes and future direction of HNSCC are significantly impacted by the presence of oncogenic human papilloma virus (HPV) infection. Our analysis revealed no significant variations in the levels of *JUN* mRNA between patients who tested positive or negative for the p16 protein (Figure S3). These findings suggest that c-Jun expression is elevated in tumors compared to normal tissue, irrespective of clinical parameters or the underlying cause of the tumor.

### A-DiFiD in combination with cisplatin potentiates HNSCC cytotoxicity

**Figure 6:**
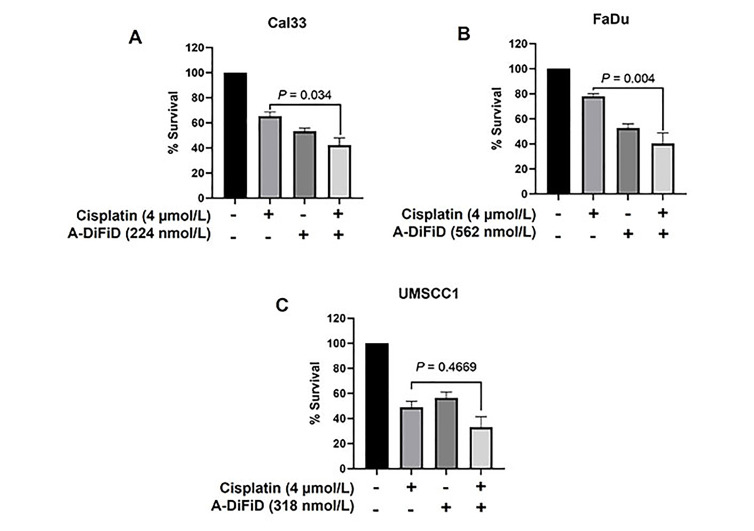
A-DiFiD in combination with cisplatin potentiates HNSCC cytotoxicity. Survival analysis of cisplatin (4 µmol/L), A-DiFiD (IC_50_), or combination treatment on (A) Cal33; (B) FaDu; (C) UMSCC1. Error bars represent ± SEM

Cisplatin, a chemotherapy medication that utilizes platinum, is commonly used as the standard treatment for HNSCC [37]. Nevertheless, despite advancements in therapeutic approaches, overcoming tumor resistance continues to be a major obstacle in effectively treating HNSCC. In our study, we evaluated the combined impact of A-DiFiD (IC_50_) and cisplatin (4.0 µmol/L) on HNSCC cell lines. The combination of A-DiFiD and cisplatin exhibited a pronounced decrease in HNSCC cell viability compared to individual treatments with cisplatin or A-DiFiD alone in Cal33 (*P* < 0.034) and FaDu (*P* < 0.004) cell lines (Figure 6A and B). However, there was only modest combinatorial effect in UMSCC1 cells (*P* = 0.4669) (Figure 6C).

### c-Jun expression decreases HNSCC survival

**Figure 7:**
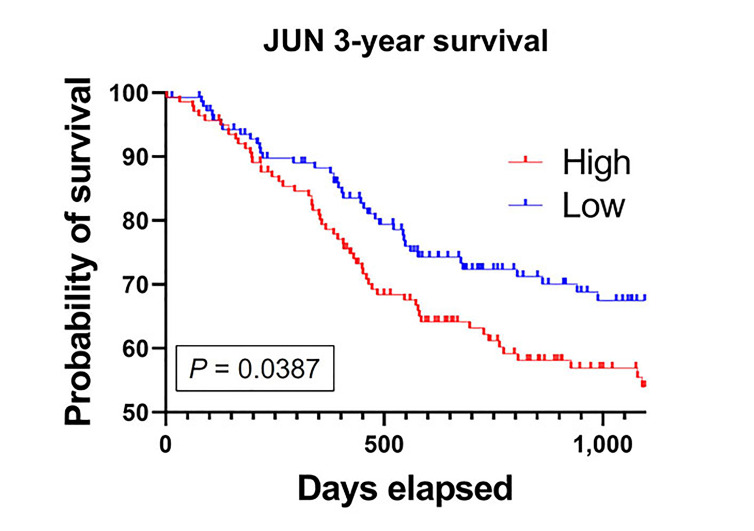
High *JUN* expression is associated with poor patient survival. Kaplan-Meier survival curve generated from TCGA HNSCC mRNA demonstrate that high *JUN* levels expressing patients have decreased survival compared to low *JUN* expressing patients (high, *n* = 141; low, *n* = 138; *P* = 0.0387)

Finally, we compared patients who had high (the top 50% of the median) *JUN* mRNA expression to low *JUN* mRNA expression. High *JUN* expressing patients had a significantly decreased 3-year survival compared to the low *JUN* expressing patients (Figure 7). This implicates c-Jun as an important clinical correlate to poor outcomes in HNSCC patients.

## Discussion

Current HNSCC therapies like cisplatin have wide ranging, deleterious and undesirable side effects [38]. Small molecule therapies that are highly cytotoxic to HNSCC cells while sparing non-cancerous tissue are highly desirable. We performed an initial screening of four small molecule analogs and found A-DiFiD to have the most potent cytotoxic effects against HNSCC (HN5 IC_50_: 245.4 nmol/L). Concurrently, Het-1A, a transformed, non-cancerous esophageal cell line, resisted A-DiFiD cytotoxicity with many folds higher IC_50_ (8.202 µmol/L) compared to HN5. Thus, A-DiFiD may be well tolerated by normal cells while exerting anti-tumor effects.

Selective molecular antagonism remains a hurdle when developing small molecule inhibitors [39]. To evaluate a possible range (37 total kinases) of possible targets we utilized a phospho-kinase array to identify the target of A-DiFiD. We found c-Jun to be the most affected target of HNSCC cells treated with A-DiFiD. We further established an attenuation of c-Jun phosphorylation starting at 3 min post A-DiFiD treatment. c-Jun expression has been known to induce cellular proliferation in human breast cancer cells [40]. Our finding corroborates their work by showing that cell proliferation does not progress in HNSCC 72 h post A-DiFiD treatment. In tandem with these results, we performed colony formation assay, which demonstrates the ability of single cells to proliferate. When HNSCC cells were pretreated with A-DiFiD at varying doses we found that single cells were unable to form colonies at IC_50_ doses. These data revealed A-DiFiD to be our best small molecule candidate with potent antitumor effects.

HNSCC distant metastatic spread significantly worsens patient prognosis, with a median survival of 10 months [41]. An early step in metastatic spread is the invasion and movement of primary tumor cells to move beyond its local site. This can be accomplished by cellular secretion of MMP that degrade the ECM boundary that confine primary tumors. Kimura et al. [42] demonstrate that c-Jun activation induced MMP secretion in osteosarcoma cells, reducing invasion. Nasopharyngeal carcinoma and glial tumor cell lines show that silencing c-Jun reduces invasion and migration *in vitro* [43, 44]. We show that A-DiFiD treatment significantly abrogated HNSCC invasion and migration. Additionally, the invasive potential of a cell is predicated on its ability to interact with the ECM and adhere to ECM substrates [45]. We performed cell adhesion/spread assay using collagen, fibronectin, vitronectin, and laminin, ECM components found in HNSCC tumors [46, 47]. A-DiFiD significantly abrogated the ability of HNSCC cells to adhere to all ECM substrates tested. This indicates that A-DiFiD reduces the tumorigenicity of HNSCC *in vitro*.

The overexpression of c-Jun has been observed in 31% of tumors in NSCLC, and it plays a role in cell proliferation, survival, and angiogenesis. Similarly, increased c-Jun expression has been detected in colorectal adenocarcinoma samples, particularly in tumor tissue rather than in normal, distant mucosa [15]. Moreover, breast cancer tumors exhibit higher levels of c-Jun expression at the invasive front [48]. Historically, transcription factors have been considered difficult to target due to the challenges associated with site-specific residue targeting [49]. However, recent advances in small molecule targeting of transcription factors have shown promising results [50]. However, there remains a paucity of small molecules targeting c-Jun across multiple cancers, and no small molecules have undergone preclinical screening in HNSCC [39]. In contrast, a c-Jun small molecule inhibitor called CC-90001 is currently being investigated in phase 2 clinical trials for idiopathic pulmonary fibrosis [27]. In our study, A-DiFiD displayed similar cytotoxicity to CC-90001.

Upstream c-Jun N-terminal kinases (JNK) directly bind and phosphorylate c-Jun. JNK1, 2, and 3 are selectively inhibited by SP600125 [26]. We found A-DiFiD displayed more potent cytotoxicity compared to SP600125. This may indicate direct inhibition of transcription factor binding is a more efficient target in the c-Jun pathway.

Indeed c-Jun is a notable oncogenic driver of multiple cancers [14–16], and more recently identified as a prognostic marker in head and neck cancer [17]. Our own interrogation of TCGA found *JUN* DNA amplification in patients with recurrent and metastatic disease was higher than patients with non-metastatic, non-recurrent primary tumors. Recurrent and metastatic HNSCC treatment is complicated by chemotherapy resistance [51]. HNSCC cisplatin resistance is promoted by the c-Jun pathway activation and c-Jun overexpression [52]. We found A-DiFiD sensitized cells to cisplatin treatment.

Additionally, we found that 3-year survival was significantly lower for HNSCC patients who expressed high levels of JUN. Interestingly, we found no difference in mRNA levels of *JUN* between patient and normal samples. However, CPTAC investigation revealed patient tumors had elevated c-Jun protein. Further, there was a sustained protein elevation across all tumor stages compared to normal, implying c-Jun may be responsible for early tumorigenesis and continued late neoplastic disease. Thus, c-Jun represents an under-targeted, yet important driver and marker of HNSCC progression.

Targeting c-Jun specifically presents certain clinical challenges that need to be addressed. This is because inhibiting c-Jun could impact cellular proliferation and turnover, resulting in delayed wound healing and potential gastrointestinal disturbances. Furthermore, c-Jun plays a crucial role in axonal regeneration in the brain [53], suggesting that targeting c-Jun may lead to neurological side effects.

However, despite these challenges, the development of a small molecule like A-DiFiD that selectively targets c-Jun while maintaining low cytotoxicity to non-cancerous cells is an exciting advancement in the field of oncogenic transcription factors. In our study, we demonstrate that A-DiFiD effectively targets c-Jun, inhibiting its *in vitro* functions in HNSCC. These findings highlight the potential of A-DiFiD as a promising therapeutic agent and warrant further investigation for its *in vivo* and clinical applications.

## Abbreviations

A-DiFiD: acryl-3,5-bis(2,4-difluorobenzylidene)-4-piperidone
ATR: attenuated total reflectance
c-Jun: cellular JUN proto-oncogene, activator protein 1 transcription factor subunit
CPTAC: Clinical Proteomic Tumor Analysis Consortium
DiFiD: 3,5-bis(2,4-difluorobenzylidene)-4-piperidone
DMEM: Dulbecco’s Modified Eagle Medium
DMSO: dimethylsulfoxide
ECM: extracellular matrix
EtOH: ethanol
HCl: hydrochloric acid
HNSCC: head and neck squamous cell carcinoma
IC_50_: half-maximal drug inhibitory concentration
JUN: JUN proto-oncogene, activator protein 1 transcription factor subunit
m.p.: melting points
m/z: mass/charge
m: minute
MMP9: matrix metalloprotease 9
NMR: nuclear magnetic resonance
p-c-Jun: phospho-cellular JUN proto-oncogene, activator protein 1 transcription factor subunit
RPKM: reads per kilobase million
SEM: standard error of the mean
TCGA: The Cancer Genome Atlas

## Supplementary materials

The supplementary material for this article is available at: https://www.explorationpub.com/uploads/Article/file/1002184_sup_1.pdf.

## Declarations

### Copyright

© The Author(s) 2023.
