# Supplementary material for: Acryl-3,5-bis(2,4-difluorobenzylidene)-4-piperidone targeting cellular JUN proto-oncogene, AP-1 transcription factor subunit inhibits head and neck squamous cell carcinoma progression
Source: Explor Target Antitumor Ther. 2023 Oct 31;4(5):1104–21. doi: 10.37349/etat.2023.00184 (PMC10651473; doi:10.37349/etat.2023.00184)
Supplement: Supplementary file 1 [file 1002184_sup_1.pdf]

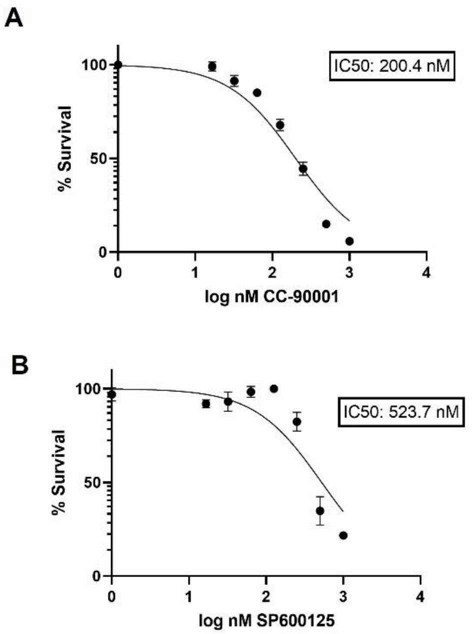


**Figure S1.** A-DiFiD demonstrates comparable cytotoxicity to existing c-Jun inhibitors. Small molecule inhibitors (A) CC-90001 and (B) SP600125 inhibitory concentration 50 ($IC_{50}$) on HN5 HNSCC cell line expressed by percent survival

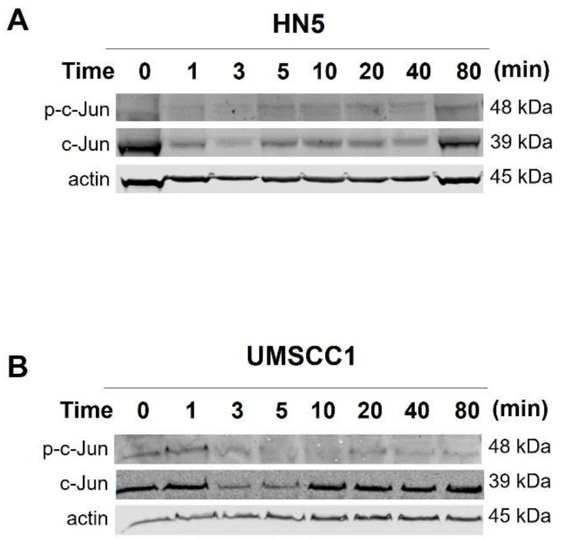


**Figure S2.** A-DiFiD attenuates c-Jun levels in HNSCC. HNSCC cells ($2 \times 10^5$ cells) HN5 (A) and UMSCC1 (B) were treated with $IC_{50}$ concentrations of A-DiFiD and protein lysate collected over various time points

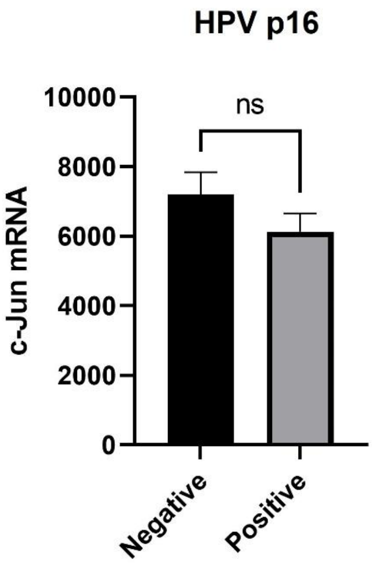


**Figure S3.** HNSCC HPV positivity does not affect c-Jun expression. JUN mRNA levels from TCGA (Firehose Legacy) as shown in transcripts per million
